# Stream benthic macroinvertebrates abundances over a 6-year monitoring period of an Italian glacier-fed stream

**DOI:** 10.3897/BDJ.7.e33576

**Published:** 2019-03-06

**Authors:** Alberto Scotti, Ulrike Tappeiner, Roberta Bottarin

**Affiliations:** 1 Institute for Alpine Environment, EURAC Research, Bolzano, Italy Institute for Alpine Environment, EURAC Research Bolzano Italy; 2 Institute of Ecology, University of Innsbruck, Innsbruck, Austria Institute of Ecology, University of Innsbruck Innsbruck Austria

**Keywords:** macroinvertebrates, community structure, biodiversity, stream, ILTER, Alps, mountains, climate change, biomonitoring, insects, Saldur

## Abstract

**Background:**

Aquatic macroinvertebrates are widely used as bioindicators for water quality assessments involving different kinds of disruptive factors, such as hydrological regime variations or pollutant spills. Recently, they demonstrated to be effective in monitoring effects of climate change in alpine stream and rivers. Indeed, since the distribution of macroinvertebrates in glacier-fed streams has been succesfully investigated and described by several authors, the discrepancy in presence/absence and quantity of specific taxa from the established models may represent an early warning of the effects of climatic changes occurring in alpine riverine ecosystems.

**New information:**

Together with the present paper, we provide a dataset covering a period of 6 years (2010-2015) sampling of aquatic macroinvertebrates along a longitudinal transect of a glacier-fed stream located in the Italian Alps, inside the International Long Term Ecological Research (ILTER) macrosite of Matsch|Mazia (IT-25). Data were collected during the glacial melt period (April - September), with monthly resolution. Owing to the unique temporal resolution of the dataset, we aim to produce a reliable tool (i.e. reference point) for future ecological assessment on the same stream, but also to similar streams worldwide.

## Introduction

Aquatic macroinvertebrates are commonly used for biomonitoring of lotic environments (Resh 2008). The main reasons for the successful utilisation of aquatic macroinvertebrates as bioindicators are their ubiquity, their generally sedentary behaviour associated with relatively long life cycles, and the high diversity (Abel 2014). As a result, macroinvertebrates allow for detailed, long-term analyses of the quality of the freshwater environment, potentially affected by different factors (e.g. discharge variations, pollutant spills) (Rosenberg and Resh 1993).

Recently, aquatic macroinvertebrates have been suggested to be used as sentinels for climate change, especially in relation to glacier retreat (Jacobsen et al. 2012, Khamis et al. 2014). Indeed, a very recent study found a direct relationship between the loss of insects in alpine riverine environments and the consequences of the changing climate (Giersch et al. 2017). In greater detail, forecasted trends in climate for glacier-fed rivers in the upcoming years suggest severe disruptions of the thermal and hydrological regime and of the sediment budget (Huss and Hoch 2018).

In this context, characterisation of abiotic and biotic factors (i.e. macroinvertebrates) in alpine streams and rivers have been of interest to ecologists for long time (e.g. Ward 1994, Milner et al. 2010). In particular, several studies have defined the distribution and diversity of benthic macroinvertebrate communities along longitudinal transects of several glacier-fed streams (summarised by Milner et al. 2001). Nevertheless, the temporal resolution of these data is often quite limited, generally not allowing long term monitoring of the study sites.

Here, we provide a dataset that contains aquatic macroinvertebrate records collected in 6 consecutive years along a longitudinal transect of the Saldur stream, an almost pristine glacier-fed small river located in the Italian Central Eastern Alps. The Saldur stream has been part of the International Long Term Ecological Research (ILTER) network since 2014 (site code: LTER_EU_IT_100). Our purpose is to release a valuable dataset that has been only partly explored by the studies carried out by Rogora et al. (2018) and Scotti et al. (2019). Moreover, since no similar datasets have been previously published, it constitutes a reference point and a relevant tool of comparison for aquatic ecologists eager to analyse the effects of climate change in other similar glacier-fed streams, given the widely reported constant distribution of aquatic macroinvertebrates found in this freshwater habitat (Milner et al. 2001).

## Sampling methods

### Study extent

**Figure 1.**
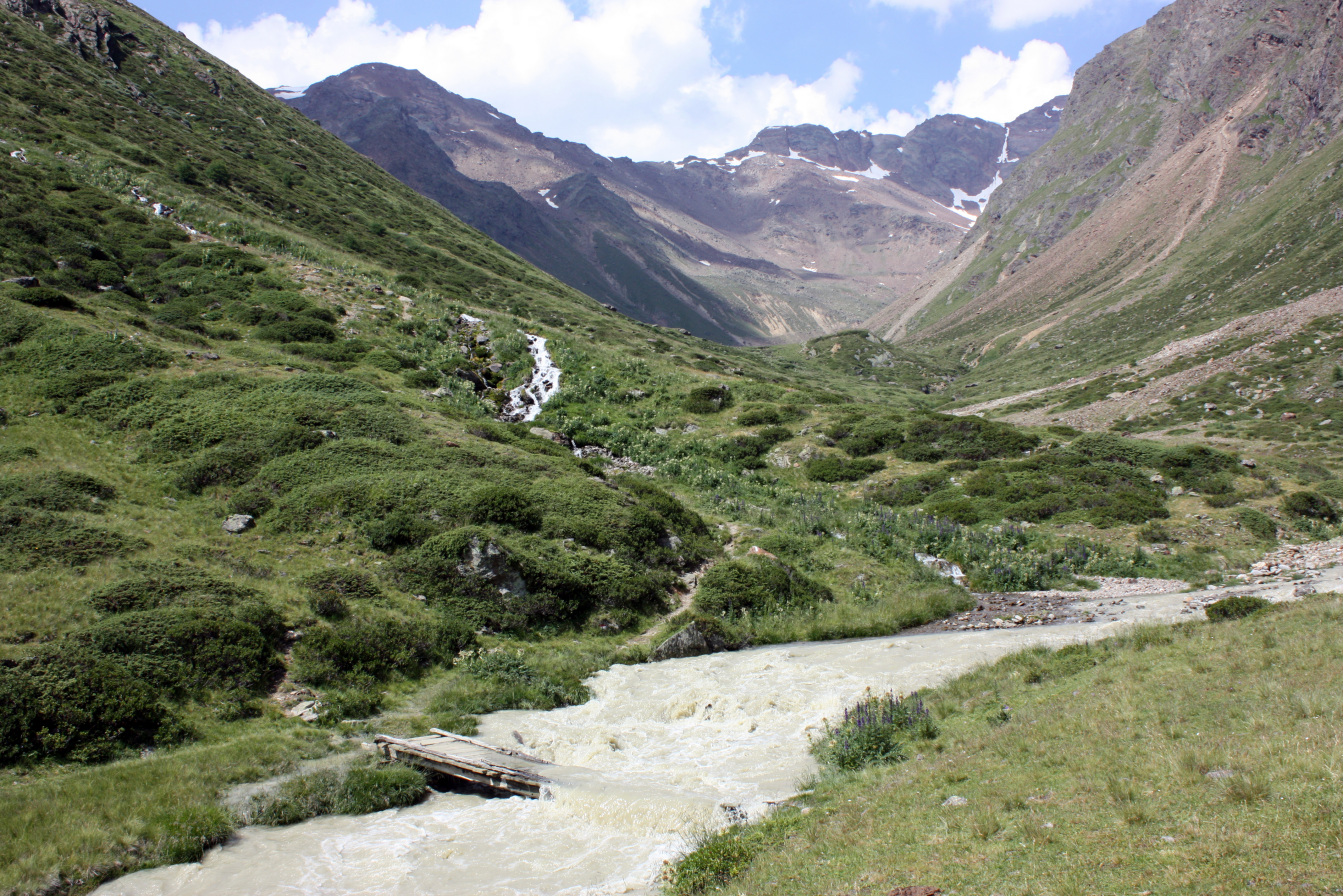
Saldur stream in July 2015, during the glacial melting period. Distance from the glacial source of the shown location is about 2 km. Picture: R. Bottarin.

Data were collected inside Matscher valley (South Tyrol, Italy; 46°N, 10°E), whose surface almost completely overlaps the Saldur stream catchment (101 km^2^) Fig. 1.

### Sampling description

For each sampling occasion at each site, we randomly collected 12 Surber samples (0.0506 square metres, mesh size 500 µm) covering all the main substrate typologies present, that were previously examined and estimated. All samples were labelled and preserved in the field with 70% ethanol. Once in the laboratory, the samples were sorted and identified.

## Geographic coverage

### Description

Saldur stream, Matscher Valley, South Tyrol (Autonomous Province of Bolzano/Bozen), Italy.

Distance from glacial source, geographic coordinates, elevation, mean stream width and depth, and size of the catchment, calculated for each sampling site, are reported in Table 1.

## Taxonomic coverage

### Description

All the sampled invertebrate organisms were considered in the study. Macroinvertebrates were identified to family or genus level referring to Sansoni and Ghetti (1998).

## Temporal coverage

### Notes

Sampling campaigns were carried out in 6 consecutive years (2010 - 2015), during the glacial melt period (April - September). Depending on the specific years, 5 to 6 samples per year were collected, thus keeping a monthly resolution for the dataset.

## Usage rights

### Use license

Other

### IP rights notes

CC-BY-NC: Creative Commons Attribution-NonCommercial 4.0 International

## Data resources

### Data package title

Bottarin, Roberta; Scotti, Alberto (2019): Abundances of stream benthic macroinvertebrates during a 6-year monitoring period of a glacier-fed stream in South Tyrol, Italy. *Eurac Research, PANGAEA*, https://doi.pangaea.de/10.1594/PANGAEA.897915

### Resource link

https://doi.pangaea.de/10.1594/PANGAEA.897915

### Number of data sets

1

### Data set 1.

#### Data set name

Saldur_invertebrates_abundance

#### Data format

tab-delimited text

#### Number of columns

9

#### Download URL

https://doi.pangaea.de/10.1594/PANGAEA.897915

#### Description

Stream benthic macroinvertebrates have been collected for a period of 6 consecutive years (2010-2015, from April to September, during the glacial melting) along a longitudinal transect of the Saldur stream, a near pristine glacier-fed stream located in South Tyrol, Italy (46°N, 10°E), part of the International Long Term Ecological Research (ILTER) network (site code: LTER_EU_IT_100).

Organisms were collected through Surber samplings (0.0506 square metres, mesh size 500 µm) in 3 sites at different elevation and distance from the glacial source: Saldur 1 (2,030 m a.s.l.; 4.962 km), Saldur 2 (2,016 m a.s.l.; 5.325 km), Saldur 3 (1,645 m a.s.l.; 11.123 km).

Dataset contains 1901 records, metadata as header.

**Data set 1.** 

Column label	Column description
Event	Sampling site
Latitude	Latitude (DD)
Longitude	Longitude (DD)
Date/Time	Date of sampling
ID	Number of consecutive record
Order	Macroinvertebrate order
Family	Macroinvertebrate family
Genus	Macroinvertebrate genus (if available)
Macrof abund [#/m**2]	Abundance of macroinvertebrates [organisms per square metre]

## Figures and Tables

**Table 1.** Geographical and local characteristics for each of the monitored sampling sites: latitude and longitude (DD), elevation (m a.s.l.), mean stream width and depth (m), catchment area (km^2^).

**Site**	**Distance from glacial source (km)**	**Latitude (DD)**	**Longitude (DD)**	**Elevation (m a.s.l.)**	**Mean stream width (m)**	**Mean stream depth (m)**	**Catchment area (km^2^)**
Saldur 1	4.962	46.743549	10.70131	2,030	6	0.4	19.7
Saldur 2	5.325	46.742365	10.699572	2,016	8	0.3	25.6
Saldur 3	11.123	46.711499	10.647516	1,645	6	0.4	61.5
